# Feasibility of Agricultural Biomass Fly Ash Usage for Soil Stabilisation of Road Works

**DOI:** 10.3390/ma12091375

**Published:** 2019-04-28

**Authors:** Ivana Barišić, Ivanka Netinger Grubeša, Tihomir Dokšanović, Berislav Marković

**Affiliations:** 1 Faculty of Civil Engineering and Architecture Osijek, Josip Juraj Strossmayer University of Osijek, Vladimira Preloga 3, 31000 Osijek, Croatia; nivanka@gfos.hr (I.N.G.); tdoksanovic@gfos.hr (T.D.); 2 Department of Chemistry, University of Osijek, Cara Hadrijana street 8/A, 31000 Osijek, Croatia; bmarkovi@kemija.unios.hr

**Keywords:** agricultural biomass ashes, stabilized soil, road construction, strength, elastic properties, 3D digital image correlation technique

## Abstract

Agricultural biomass ash is a waste material produced by incineration of residue from fields after harvesting crops. The use of agricultural biomass in industry produces large quantities of ash that represent an ecological problem. Another ecological problem is the dependency of road building on natural materials, which has been traditionally used for all pavement layers. Today, roads are built on less accessible and suitable terrains, increasing the need for improving the mechanical characteristics of locally available materials by various means of stabilisation. Within this research, three agricultural biomass fly ashes are used as lime substitutes for hydraulically stabilised soil. The purpose of this research is evaluation of potential use of agricultural biomass fly ash for the soil stabilisation of road works, i.e., for embankment and subgrade purposes. The results indicate that there is a potential of using barley, sunflower seed shells and wheat fly ash as lime substitutes in the soil stabilisation of road works. The strength characteristics of stabilised soil incorporating biomass fly ash are highly dependent on its chemical composition. Using a three-dimensional digital image correlation technique, it is concluded that the elastic properties of stabilised soil correlate to a fracture mechanism that can be efficiently defined by this modern research tool.

## 1. Introduction

In Croatia, 52% of the total territory is agricultural land, with ~80% of the arable surface under maize, wheat, soybean, sunflower, rapeseed, grapevine, olive, apple and plum cultivation [1]. This presents great potential for agricultural biomass usage in energy production but there is also a need for sustainable waste management, since significant amounts of bio-ash will be generated.

Agricultural biomass is a residue in the fields after harvesting crops that is then used to produce energy in biomass power plants. Although a significantly lower quantity of ash is generated during agricultural biomass combustion compared to coal, this ash is not being suitably managed and new applications for it need to be found. Currently, waste from biomass incineration is being disposed of at landfills, on farmland or in forests, most often without any control, which can cause environmental pollution and potential human health risks [2]. 

Road construction is the branch of civil engineering that is most dependent on natural material availability. Simultaneously, increases in traffic loads and the need for building roads on less accessible and suitable terrain result in a need to find new ways of locally available material usage, as well as improving their mechanical characteristics. Consequently, the ash generated from agricultural biomass incineration is being studied as a material for all pavement layers. Depending on its characteristics, ash can be utilised as a filler, as a replacement for small aggregate fractions, as a binder itself when it contains active minerals (e.g., lime, calcium and magnesium silicate or alumina silicates), resulting in hydraulic binding, or as a binder supplement or addition when it contains pozzolanic minerals (e.g., glass, Portland, gypsum or clay minerals), which in combination with other materials leads to a pozzolanic reaction [3]. For wearing course construction, biomass ash has been investigated for both asphalt [4,5,6] and concrete [7,8] pavements. However, for both pavement systems, a good quality subgrade is of high importance. Locally available soil is often not suitable for subgrade or embankment construction, so different ways of stabilisation are used, most often by lime or cement. 

Due to its potential pozzolanic and hydraulic characteristics, there is a high potential of using bio-ash in soil stabilisation for the specified purposes. The use of bottom ash from biomass (olive) combustion reduces the expansion of expansive soils to the same extent as from treatment with lime, as presented in [9]. Research results indicate that 6–8% cement and 10–15% rice husk ash are optimal additions for residual soils from the viewpoints of plasticity, compaction, strength characteristics and cost [10]. The stabilisation of alluvial soil by biomass ashes from rice husk and sugar cane bagasse results in a plasticity index decrease with an increase in the proportion of ash from 2.5% to 12.5%, with the optimal ash content for stabilisation reported to be 7.5% [11]. Admixing of rice husk ash, bagasse ash and rice straw ash with soil results in a higher optimal moisture content as the dosages of stabilisers increase [12]. The addition of the same ash to clayey soil at a concentration of 20–25% also increased the California Bearing Ratio (CBR) values. 

Rice husk and sugarcane bagasse-based mixed biomass ash combined with hydrated lime as an activator in clay resulted in an increase in compressive strength, as described in [13]. Similar results are presented in [14], where the addition of bagasse ash to expansive soils results in CBR, compressive strength and maximum dry density increases, as well as a swelling decrease [15]. Sugarcane straw ash can also be an effective stabiliser for improving the geotechnical properties of lateritic soil samples [16]. The combination of wheat husk and sugarcane straw ash also positively influences the geotechnical properties of soil [17]. Soil admixtures with coal fly ash and rice husk ash have the potential to improve soil resistance to permanent deformation [18]. Biomass furnace ash from agricultural olive residues can also be used as a filler material in road embankments [19]. In contrast, biomass fly ash of olive waste used in [20] was found to be the least effective additive in the stabilisation of marl soil, which indicates that its effectiveness could depend on the type of soil to be treated.

Thus, the aim of this study is to identify possible applications of biomass ashes in order to promote sustainable energy production from which it originates and to preserve natural resources and energy needed for lime production. Namely, energy generated from the biomass production is currently the fourth most common energy source in the European Union [1] and large quantities of waste biomass ash are generated. Before recycling of biomass ashes as construction materials, detailed investigation need to be conducted, demonstrating its acceptable level of performance and economical comparability to traditional materials. Therefore, the purpose of this research is to define basic characteristics of agricultural biomass ash for its potential earthwork application in road construction. This article reports an experimental study of the properties of three biomass ashes used as additives to lime stabilised low bearing soil for embankment and subgrade purposes.

## 2. Materials and Methods

### 2.1. Raw Materials

**Figure 1:**
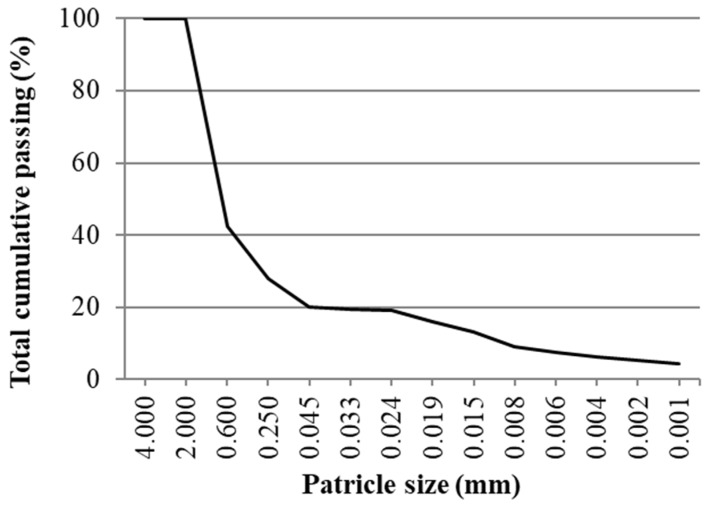
Soil particle size distribution curve.

The size distribution of used soil was determined according to standard EN ISO 17892-4 by combination of sieving and hydrometer methods. The particle size distribution curve is presented in Figure 1 and the density of soil used was 2.74 kg/dm^3^. Specific surface area (SSA) of used soil determined by the Brunauer, Emmett and Teller (BET) method according to standard ISO 9277 is 9760 cm^2^/g.

The liquid and plastic limits of the used soil, determined according to standard EN ISO 17892-12, were 34.5% and 21.9%, respectively, with a plasticity index of 12.5%. It was classified as low plasticity clay-CL according to the Unified Soil Classification System (USCS). The optimal water content and maximal dry density, determined by standard EN 13286-2, were 13% and 1.80 g/cm^3^, respectively. 

For soil stabilisation, CL 80 S hydrated calcium lime was used according to EN 459-1, with a density of 2.65 kg/dm^3^. SSA of used lime determined by the BET method according to standard ISO 9277 is 16,671 cm^2^/g.

As a binder substitute, three biomass fly ashes were used. The oil factory Čepin uses sunflower seed shells as a fuel during sunflower oil production. Biomass is burned within the furnace, in a hot-air stream, and the biomass ash produced for the purpose of this research was collected from a specialized landfill within factory premises. In order to test some new energy resources, it has been tried as a replacement for sunflower seed shells by barley and wheat straws. Fly ash from sunflower seed shells (S), barley (B) and wheat straws (W) from this factory was used in this study, with the chemical composition of the used ashes determined in accordance with ISO/TS 16996:2015 presented in Table 1. The densities of the S, B and W ashes were 2.26, 2.23 and 2.36 kg/dm^3^, respectively, tested according to standard EN 1097-7. The SSA for the S, B and W ashes was 33,740, 34,080 and 36,850 cm^2^/g, respectively, determined by the BET method according to standard ISO 9277.

### 2.2. Sample Preparation and Strength Tests

The optimal lime and ash portions were determined by standard ASTM D 6276-99a, measuring the pH values of soil-lime and soil-lime-ash mixtures with various content ratios. It was determined that the optimal lime content is 7% of the total dry mass of soil and the optimal lime/ash ratio is 80%/20%. After defining the optimal stabilised soil composition, the maximum dry density (MDD) and optimal water content (OWC) were determined according to standard EN 13286-2. The specimens were prepared at their respective OWC and maximal dry density (MDD), measured after compaction 100 mm in diameter and 200 mm in height. Prepared specimens were cured for 28 days in a temperature and moisture controlled chamber (20 °C and 60% relative humidity). The compressive strength test (according to standard EN 13286-41) and the 3D digital image correlation (DIC) were determined for these specimens. The CBR and linear swelling were determined according to standard EN 13286-47.

### 2.3. Elastic Properties-3D Digital Image Correlation

**Figure 2:**
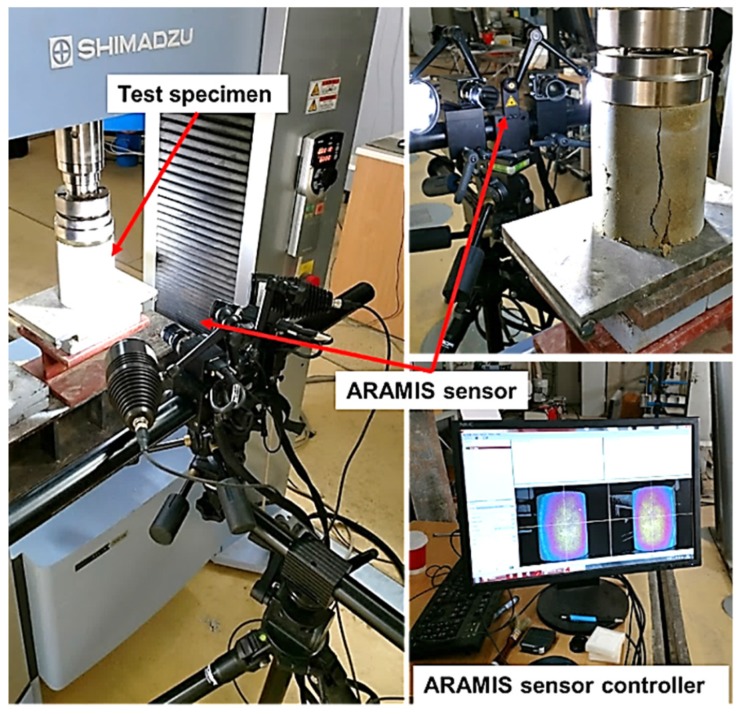
Test setup for 3D digital image correlation (DIC) measurements.

DIC is a non-destructive, non-contact method used for determination of loaded object surface deformation, i.e., it allows us to track displacements in a field of view of applied cameras (Figure 2). It can be utilised using a single camera (2D) or two camera (3D) setup, and further details on this method and its potential application are presented in [21]. Spatial DIC measurements for the purpose of this study were implemented using a GOM Aramis 3D optical deformation analysis system, along with its corresponding software package. Specimens were monitored during compressive testing, with the force data being supplied to the DIC system via an output channel of the utilised Shimadzu AG-X universal testing machine, connecting deformation stages to corresponding frames. The system was set to capture images with a frequency of 4 Hz, per camera, which was suitable to obtain more than 50 images (data points) in the elastic range. The analysis of obtained recordings enabled the determination of elastic modulus in accordance with EN 13286-43 and insights into the development of fracture mechanisms.

Although classical, contact-based, instrumentation can provide data regarding the elastic modulus, such data is more prone to errors due to problems with adequate contact, concentration of deformation, gauge length influence, and so on. Additionally, such point-based instrumentation cannot provide adequate information on the fracture mechanism, i.e., deformation concertation and propagation. By tracking the entire field of view, i.e., the entire visible surface, deformation results are more reliable and an adequate insight into the phenomena of deformation distribution and redistribution with load increase can be obtained. These insights can be of great importance when assessing ductility and possible mechanisms of fracture for a certain material type.

## 3. Results and Discussion

### 3.1. Geotechnical Characteristics

The addition of lime and fly bio-ashes S, B and W resulted in plasticity index decreases of 10.77%, 11.09%, 11.20% and 10.60%, respectively, compared to the plasticity index of pure soil (CL) of 12.5%. Compared to sole lime, the addition of wheat straw fly ash presents an additional reduction in plasticity index. This reduction in plasticity index is in line with results presented in [11] and results in a soil improvement in terms of higher stability and less swelling affinity. This is also confirmed by a linear swelling test conducted in parallel to the CBR testing. The reductions in linear swelling with the addition of lime, S, B and W fly ash compared to non-stabilised soil (swelling of 5%) were 71.5%, 57.3%, 44.7% and 24%, respectively. 

**Figure 3:**
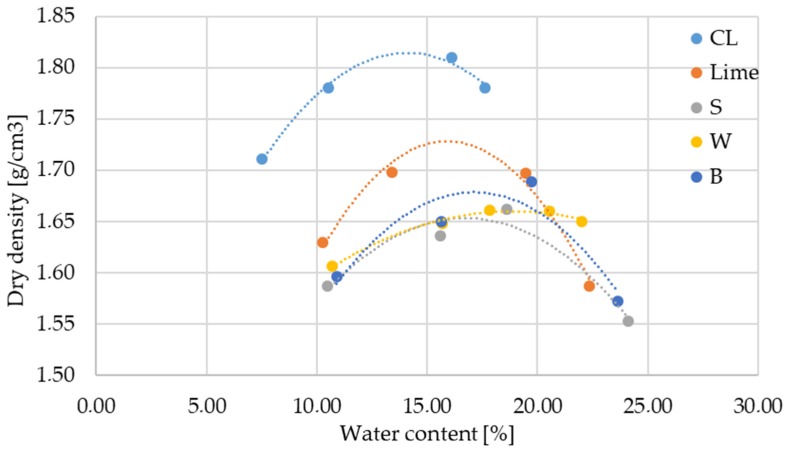
Standard Proctor compaction test results.

**Figure 4:**
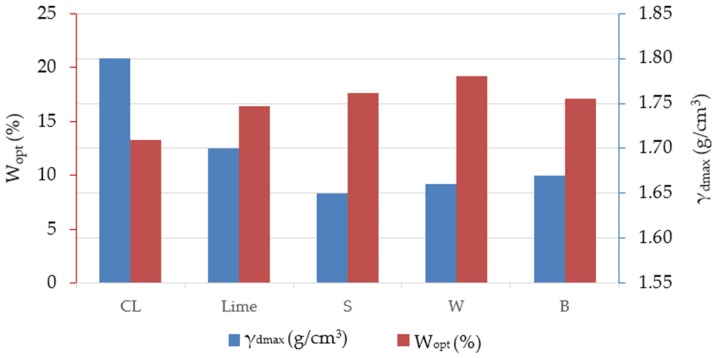
Compaction characteristics.

The results for the optimal water content and maximal dry density measurements are presented in Figure 3 and Figure 4. All tested mixtures have similar standard Proctor compaction curves. All mixtures show similar sensitive to moisture deviation, with the exception of wheat straw fly ash, which shows the lowest sensitivity due to its plane Proctor compaction curve [9].

The addition of bio-ash results in an increase in the OWC and a decrease in the MDD. The addition of wheat straw fly ash results in the highest OWC (19.18%) and a significantly lower MDD compared (1.66 g/cm^3^) to pure soil (13.30% and 1.80 g/cm^3^, respectively). This also presents an improvement in soil characteristics, since earth works may be done with more moisture in soil during the rainy season. The decrease in MDD may be due to the low specific gravity of bio-ash replacing higher specific gravity lime [11,17], which is also confirmed by measuring SSA by the BET surface analyses. All used ashes have significantly higher SSA compared to lime, as presented in Section 2.1. An increase in OWC is attributed to the pozzolanic reaction between fly ash and soil constituents, and the extra water required for higher fineness in fly ash (highest SSA and SiO_2_ for W ash) and the subsequent enhanced hydration [22]. The decrease in density was directly attributed to the flocculation/aggregation and the formation of cementitious products [12]. The reduction in MDD is attributed to the lower specific gravity of fly ash and lime compared to compacted soil [15].

### 3.2. Strength Characteristics

**Figure 5:**
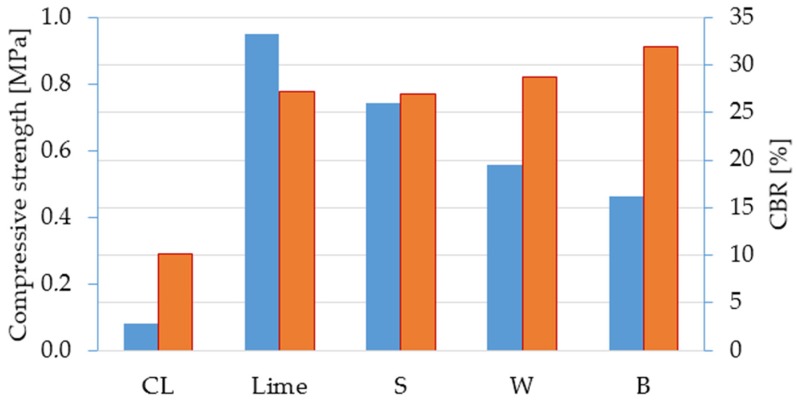
Results of CBR and compressive strength tests.

The results of uniaxial compressive strength testing are presented in Figure 5. It can be seen that addition of bio-ash results in an increase in compressive strength. The highest compressive strength is obtained for barley fly ash, with a 17% increase in comparison to pure lime stabilised soil and a 213% increase in comparison to non-stabilised soil. As presented in Table 1, barley fly ash has the highest K_2_O content, with a notably high SiO_2_ content, which contributes to the development of strength through an alkali silicate (K_2_O) and pozzolanic reaction (SiO_2_, particularly its reactive form [23]). Although sunflower seed ash has the highest CaO and MgO content, the determined compressive strengths were the lowest, which could be attributed to low SiO_2_ and high free or reactive CaO content [23].

The addition of bio-ash unexpectedly resulted in the soaked CBR values decreasing in comparison to lime stabilised soil. The lowest CBR value is obtained with the addition of barley fly ash. The alkali silicate reaction within mixtures with lime-barley fly ash is not activated because K_2_O from fly ash is dissolved in water under soaked conditions (sample immerged in water for 96 h prior to CBR testing). As reported in [24], the presence of K_2_O in fly ash increased its dissolution property, and a similar report is presented in [25]. Additionally, under soaked conditions, the destruction of capillary forces is attributed to being one of the reasons for lower CBR values [26]. The affirmation of these conclusions was verified by measuring a barley fly ash mixture CBR over a three-day soaking period (instead of four as for all other samples). A CBR of 19% was obtained, significantly higher compared to the four-day soaking period of 16%. The fly ash content was determined as the mass% of total binder content. Due to the lower density and higher SSA of all used ashes compared to lime, ash occupies more space within the sample. A certain amount of fly ash may not partake in strength development reactions, reducing bonds in the soil–ash mixtures [11]. 

Within this research, the optimal fly ash content was determined as the mass percentage of lime by measuring the pH of soil–lime–fly ash mixtures, according to ASTM D 6276-99a. Due to trends in compressive strength and CBR test results, it can be concluded that this method should take into account chemical composition, density and specific surface area of all constituents in order to properly define the optimal binder content. Additionally, the optimal bio-ash content should be determined by volume percentage rather than mass percentage. 

### 3.3. Elastic Properties and Fracture Mechanism

For an effective mechanistic-based pavement design, which relies on the elastic theory, elastic mechanical properties are required, including the Young’s modulus of elasticity (E) and Poisson’s ratio (ν). The elastic modulus as a measure of soil stiffness is determined as stress to the corresponding strain ratio in the range of elastic soil behaviour. For practical design situations, various empirical correlations to CBR values are used to calculate E, as can be found in [27,28,29]. However, due to unusual aspects of behaviour and based on results obtained on bio-ash stabilised soil, as elaborated in the previous sections, E was measured during compressive strength testing. In order to obtain the most accurate results and to eliminate problems with surface conditions and adequate specimen-instrument contact throughout loading [21], rigid ring extensometers are replaced by “virtual” extensometers, i.e., DIC. Such an application highlights the primary advantages of utilising a 3D DIC system, the possibility to monitor stress and strain of an entire surface, and the possibility to gain insight into deformation distribution and redistribution with load increase. This is a new application of 3D DIC since it has been used within research of active arching effect in soil [30]. 

**Figure 6:**
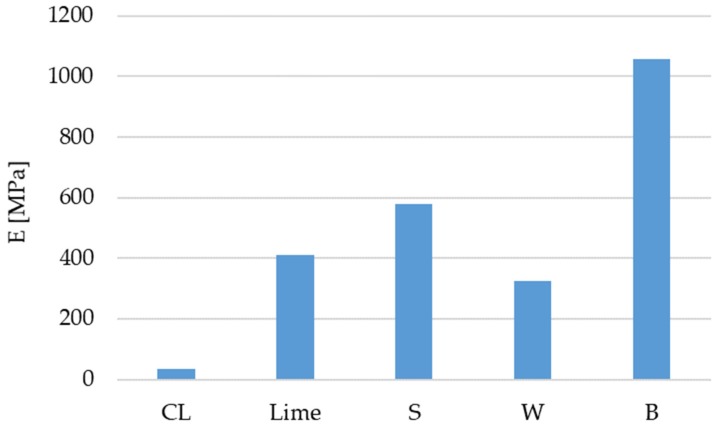
Results of modulus of elasticity test.

The results of the Young’s modulus calculation are presented in Figure 6. Among the stabilised mixtures, the one with barley fly ash has the highest E, while the lowest E is recorded for mixtures with wheat fly ash. The causality of such results is directly associated with the analysis of fracture mechanisms for each of the mixtures.

**Figure 7:**
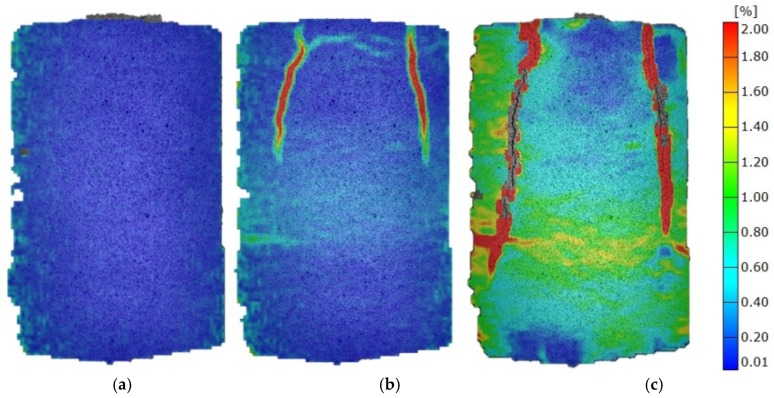
Mises strain at various load stages of mixtures with barley fly ash. (**a**) 0.3 F_max_; (**b**) 0.6 F_max_; (**c**) 1.0 F_max_.

Mixtures with barley fly ash exhibit a high degree of homogeneity, which is evident from the absence of concentrations of deformation at 30% of maximum force (F_max_), and successful redistribution of strain to the rest of the sample, even when clear indicators of fracture mechanism formulation at 60% of F_max_ are revealed. Areas of concentrated deformation in the form of vertical cracks open slowly with load increases and at loads closer to F_max_ these cracks are very clear and have a progressive propagation until fracture (Figure 7). High homogeneity could be the result of barley fly ash adequate SSA and highest MDD comparing to other tested ashes but also the most proper way of sample preparation.

**Figure 8:**
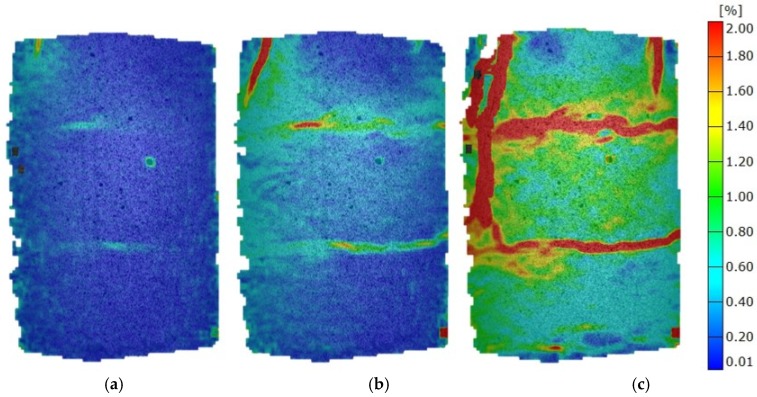
Mises strain at various load stages of mixtures with wheat fly ash. (**a**) 0.3 F_max_; (**b**) 0.6 F_max_; (**c**) 1.0 F_max_.

When wheat fly ash is used at 60% of F_max_, there are no clearly defined strain concentrations, but there are horizontal areas (layers) with prominent deformations. With a load increase, these prominent deformations transform into horizontal cracks, which are additionally pronounced and intersected with vertical cracks at F_max_. Such a fracture mechanism clearly points to horizontal inhomogeneity, which is a result of sample preparation (three layers during Proctor compaction), and which is consequently reflected in the elastic modulus value being the lowest of stabilised soils (Figure 8).

**Figure 9:**
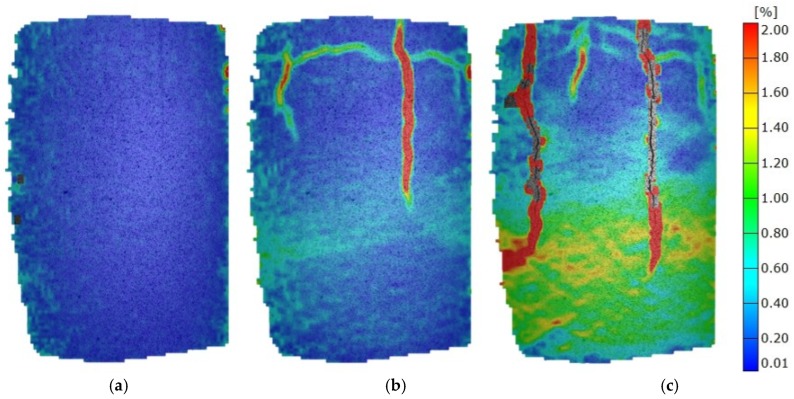
Mises strain at various load stages of mixtures with sunflower fly ash. (**a**) 0.3 F_max_; (**b**) 0.6 F_max_; (**c**) 1.0 F_max_.

Soil stabilised with sunflower shows no significant signs of dominant deformation areas at 30% of F_max_, and at 60% of F_max_, there are clear indicators of horizontal and vertical cracks (Figure 9). The fracture mechanism presents a combination of mechanisms obtained on specimens stabilised with barley and those stabilised with wheat. This coincides with the elastic modulus results of sunflower mixtures being between the other two mixtures.

**Figure 10:**
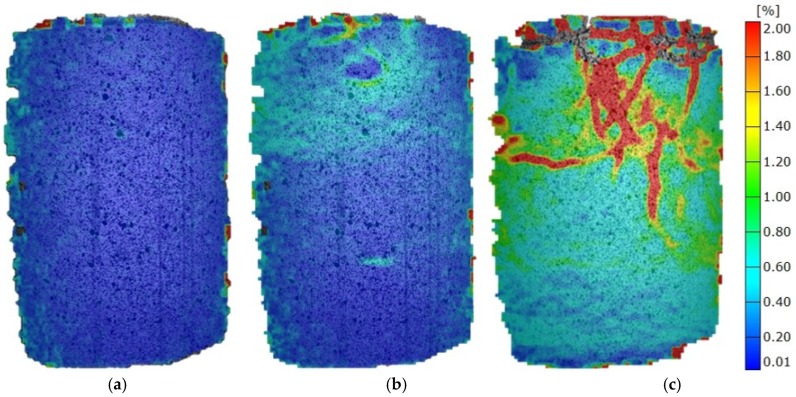
Mises strain at various load stages of mixtures with lime. (**a**) 0.3 F_max_; (**b**) 0.6 F_max_; (**c**) 1.0 F_max_.

Lime stabilised mixtures exhibit no clear localisation of deformations at 30% of F_max_, with only slight changes at 60% of F_max_ (Figure 10). At loads near F_max_, a combination of horizontal and vertical concentrations appears, which is similar to sunflower mixtures, although less emphasised.

**Figure 11:**
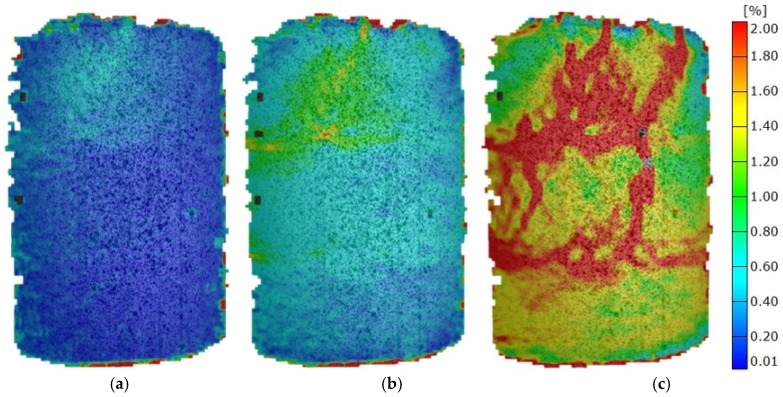
Mises strain at various load stages of mixtures with no stabilisation. (**a**) 0.3 F_max_; (**b**) 0.6 F_max_; (**c**) 1.0 F_max_.

For non-stabilised soil, failure occurs as a complete collapse of the sample with horizontal, vertical and inclined cracks along the whole sample height, with no clear fracture planes. Fields of pronounced strains can be perceived as early as 30% of F_max_ and there is a noticeable grouping of deformation in horizontal bands that coincides with the layered sample preparation procedure (Figure 11). 

**Figure 12:**
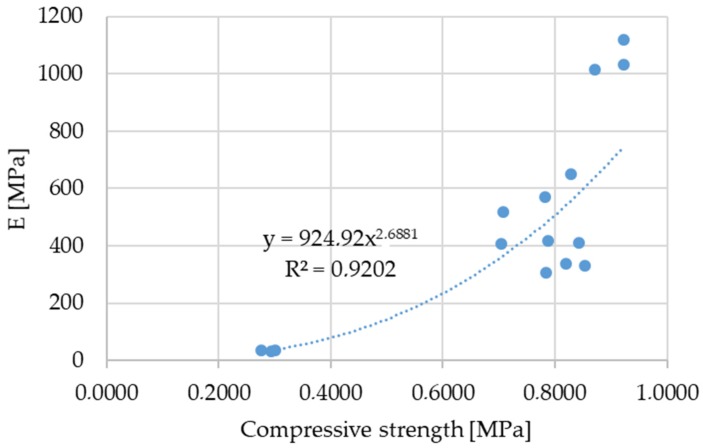
E to 28-day compressive strength correlation.

In order to predict the Young’s elastic modulus of elasticity of stabilised soil, the correlation with the 28-day compressive strength is presented in Figure 12. There is a strong correlation between E and the 28-day compressive strength. Measurements of the 28-day compressive strength as relatively simple tests could be used for E prediction as an alternative to CBR testing. However, the correlations presented here are results of research executed on a limited number of biomass ash stabilized soil mixtures. In order to set general conclusions, more tests are to be conducted on different biomass ash and soil types, and lime contents.

## 4. Conclusions

The results indicate that there is potential for using barley, sunflower seed shells and wheat fly ash as lime substitutes in soil stabilisation for road works. Based on the results, the following conclusions can be drawn:

- A lime/biomass fly ash binder improved the geotechnical characteristics of low plasticity clay by reducing the plasticity index and linear swelling and increasing the optimal moisture content.
- When evaluating the potential application of biomass fly ash as a binder substitute, its chemical composition needs to be considered.
- The addition of biomass fly ash results in a soaked CBR value decrease and a compressive strength increase.
- The strength characteristics of stabilised soil incorporating biomass fly ash are highly dependent on its chemical composition.
- The elastic properties of stabilised soil correlate to a fracture mechanism.
- Using a 3D digital image correlation technique as a modern research tool can be efficiently used for fracture mechanism and elastic properties analyses of hydraulically stabilised soil for road works.
- There is a strong correlation between Young’s modulus of elasticity and compressive strength, which can be used for its prediction for pavement design purposes.

## Figures and Tables

**Table 1:** Chemical composition of sunflower seed shells (S), barley (B) and wheat straw fly ash (W).

Oxides (mas.%)	S	B	W
P_2_O_5_	18.71	4.40	6.70
Na_2_O	<0.10	0.26	0.21
K_2_O	24.13	44.16	32.46
CaO	28.68	10.20	15.26
MgO	21.68	4.15	7.19
Al_2_O_3_	0.52	0.68	0.86
TiO_2_	0.01	0.03	0.05
Fe_2_O_3_	0.31	0.38	0.63
SiO_2_	1.96	23.38	29.49
MnO	0.08	0.03	0.05
SO_3_	3.91	12.34	7.11
